# Nitro-Grela-type complexes containing iodides – robust and selective catalysts for olefin metathesis under challenging conditions

**DOI:** 10.3762/bjoc.11.198

**Published:** 2015-10-06

**Authors:** Andrzej Tracz, Mateusz Matczak, Katarzyna Urbaniak, Krzysztof Skowerski

**Affiliations:** 1 Apeiron Synthesis SA, Duńska 9, 54-427 Wrocław, Poland; 2 Department of Bioorganic Chemistry, Faculty of Chemistry, Wroclaw University of Technology, Wybrzeże Wyspańskiego 27, 50-370 Wrocław, Poland

**Keywords:** green solvents, macrocyclization, metathesis, ruthenium

## Abstract

Iodide-containing nitro-Grela-type catalysts have been synthesized and applied to ring closing metathesis (RCM) and cross metathesis (CM) reactions. These new catalysts have exhibited improved efficiency in the transformation of sterically, non-demanding alkenes. Additional steric hindrance in the vicinity of ruthenium related to the presence of iodides ensures enhanced catalyst stability. The benefits are most apparent under challenging conditions, such as very low reaction concentrations, protic solvents or with the occurrence of impurities.

## Introduction

**Figure 1:**
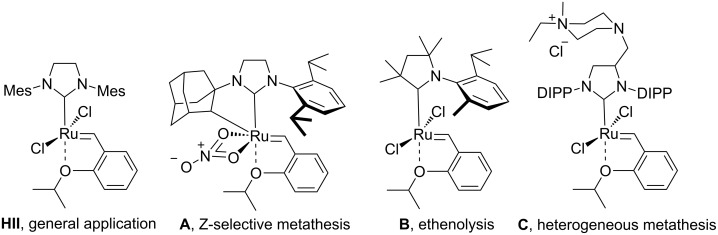
The diversity of Hoveyda-type complexes (Mes – 2,4,6-trimethylphenyl, DIPP – 2,6-diisopropylphenyl).

Olefin metathesis (OM) is a mild and versatile catalytic method which allows the formation of carbon–carbon double bonds [1]. Understanding the key events in ruthenium-catalyzed olefin metathesis [2] and developing efficient and selective catalysts [3] provides opportunities for industrial applications of this technology. In many cases, however, the achievement of high turn over numbers (TONs) requires tedious purification of starting materials and solvents. New catalysts with increased efficiency and selectivity, especially under challenging conditions, are therefore of high interest. Currently, the second generation Hoveyda-type catalysts, such as **HII** [4], **A** [5], **B** [6], and **C** [7] are considered to be the most versatile tool for OM (Figure 1).

**Scheme 1:**
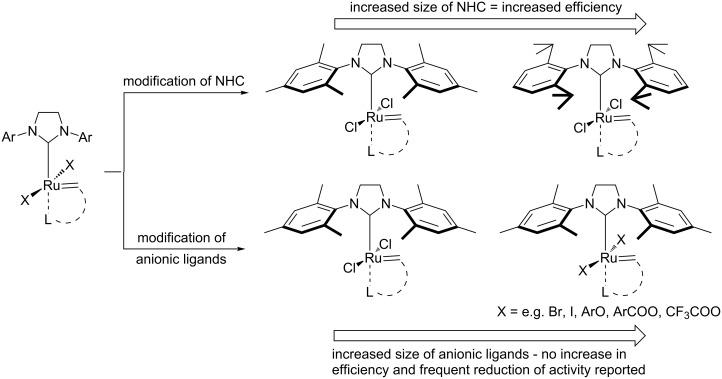
Modifications of the 2^nd^ generation alkylidene complexes.

Modifications of ligands permanently bound to the ruthenium center appear to be the most efficient methods for altering the catalyst properties. Great improvement of catalyst efficiency in the transformation of sterically non-demanding alkenes have been achieved by the replacement of the classical SIMes ligand with the bulkier SIPr ligand (Scheme 1) [8–9]. Metathesis catalysts with even larger NHC ligands have also been reported, but their syntheses require additional steps because the necessary anilines – the starting materials for the preparation of NHCs precursors – are not commercially available [10–11]. Up until now, there had been no disclosures of increased catalyst efficiency caused by the exchange of chlorides with larger anionic ligands. Grubbs et al. showed that the exchange of chlorides for bromides or iodides in the second generation Grubbs’ catalysts facilitated the initiation, but reduced the propagation rate and eventually provided no overall improvement [12]. More recently Slugovc et al. synthesized bromo- and iodo- analogues of **HII,** but no improvement was noted [13–15]. Moreover, the presence of iodide ligands reduced initiation rates for Hoveyda second generation complex bearing iodides (**HII-I2**) in ring-closing metathesis (RCM). Similarly, Schrodi and colleagues did not find any advantages for halide exchanged Hoveyda-type complexes in cross metathesis of methyl oleate with ethylene [16]. Complexes containing iodide lead to products of asymmetric OM with better enantio- and diastereoselectivity, but this came at the price of lower activity [17]. In the past few years the replacement of chloride ligands created the first *Z*-selective catalysts [18–21]. Their efficiency, however, is noticeably lower than that observed for classical complexes. The second generation indenylidene catalysts with phosphite ligand (frequently reported as “Cazin-type catalysts”) bearing mixed chloride–fluoride or difluoride anionic ligands were also reported very recently [22]. The former catalyst exhibited thermal stability and efficiency comparable with the original complex having two chlorides, while the difluoride catalyst showed low catalytic activity. Finally, alternative anionic ligands have been used in order to heterogenize catalysts, which resulted in the formation of materials with reduced activity and efficiency [23–24].

It is well recognized that the benzylidene ligand structure strongly influences initiation rates for Hoveyda-type catalysts [25]. As a consequence of the “boomerang effect”, which was recently strongly supported by Fogg et al. [26], the benzylidene ligand also most likely affects propagation rates.

In our search for active, more robust and selective catalysts, we synthesized iodide-containing nitro-Grela type catalysts. A synergistic effect of the ligands was sought: the nitro-substituted benzylidene ligand was expected to ensure fast initiation, while the bulky iodides were anticipated to provide additional stabilization of the active species.

## Results and Discussion

**Scheme 2:**
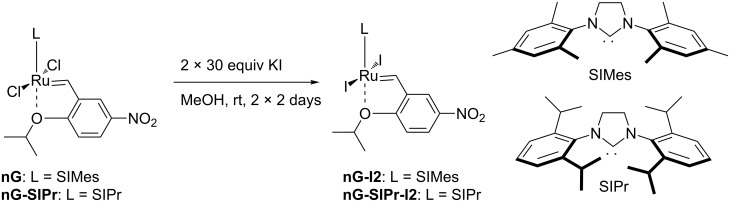
Synthesis of iodide-containing nitro-Grela type catalysts.

The new iodide-containing catalysts, **nG-I2** and **nG-SIPr-I2,** were prepared with a 93% yield from commercially available complexes, **nG** and **nG-SIPr**, and with the use of potassium iodide as the iodide anions source (Scheme 2). In the synthesis of both catalysts, the isolated material contained 99% of the expected diiodo catalyst and 1% of the “mixed halogen” complex, which was identified by field desorption mass spectrometry (FD–MS) and quantified by ^1^H NMR.

**Figure 2:**
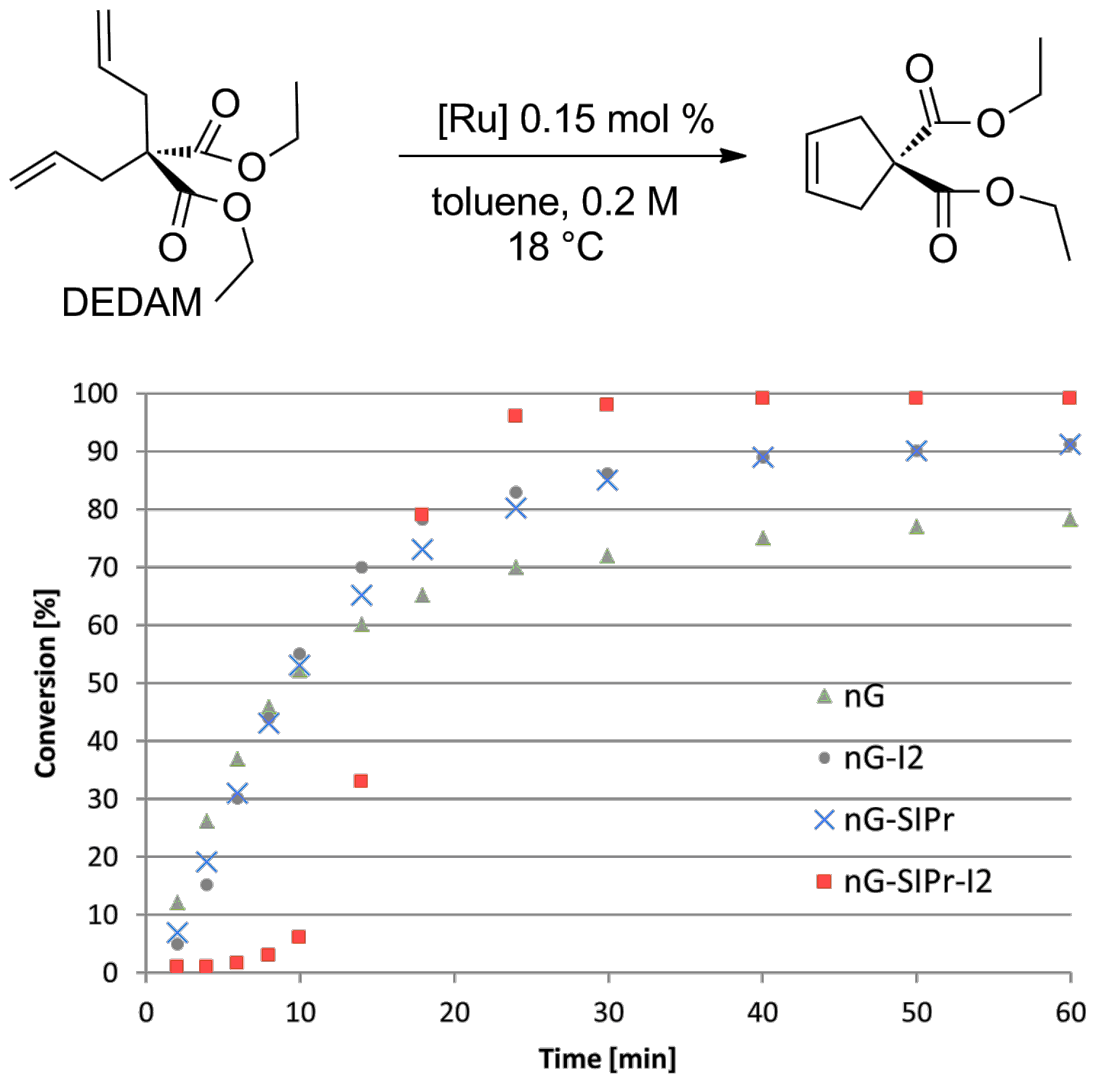
Reaction profiles for RCM of DEDAM; toluene, 0.2 M, 18 °C, [Ru] 0.15 mol %; conversion determined by GC.

In order to determine the differences in the initiation rate between the new and parent complexes, we ran the RCM of diethyl diallylmalonate (DEDAM) in toluene (C^0^_DEDAM_ 0.2 M) at a relatively low temperature (18 °C) with only 0.15 mol % of the catalyst (Figure 2). The **nG-I2** catalyst initiated slightly more slowly than the parent **nG**, but was more stable and after 1 h gave greater than a 10% better conversion of the substrate as indicated in Figure 2. The catalytic performance of **nG-I2** was almost identical to that observed for **nG-SIPr**, suggesting that the exchange of chloride with iodide can – at least for some substrates – provide similar catalyst stabilization as the introduction of a bulky NHC ligand. In the case of the most sterically crowded **nG-SIPr-I2**, initiation was delayed, but a very fast reaction propagation was observed. This catalyst was the most stable and efficient among all tested complexes.

To gain more information about the scope of application of the obtained catalysts, we carried out a set of standard RCM and CM transformations (Table 1 and Table 2). The reactions were performed in dry, degassed toluene, at 70 °C with varied catalyst loadings to demonstrate differences in their efficiencies.

The efficiency pattern observed in RCM of DEDAM was confirmed in the synthesis of five- to seven-membered, disubstituted heterocycles (Table 1, entries 1–3). Both **nG-I2** and **nG-SIPr-I2** proved to be sensitive to the steric bulk in close proximity to the double bond. Thus, RCM with substrate **7** having one double bond terminally substituted with the phenyl ring as well as the formation of the trisubstituted heterocycle **9** proceeded better with chloride-containing catalysts. When proline derivative **10** was used, the diiodo catalysts performed better than **nG** but slightly worse than **nG-SIPr**.

**Table 1:** Results of RCM reactions.^a^

Entry	Substrate	Product	Catalyst (mol %)	GC Conversion [%]

1	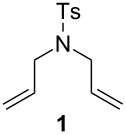	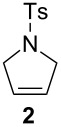	**nG** (0.0025)	32
			**nG-I2** (0.0025)	72
			**nG-SIPr** (0.0025)	85
			**nG-SIPr-I2** (0.0025)	95
2	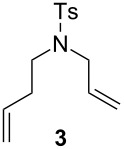	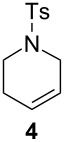	**nG** (0.003)	67
			**nG-I2** (0.003)	91
			**nG-SIPr** (0.003)	90
			**nG-SIPr-I2** (0.003)	97
3	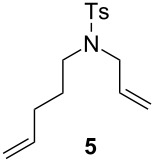	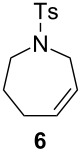	**nG** (0.0075)	57
			**nG-I2** (0.0075)	87
			**nG-SIPr** (0.0075)	86
			**nG-SIPr-I2** (0.0075)	94
4	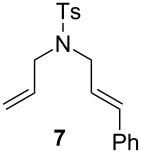	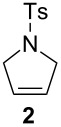	**nG** (0.015)	89
			**nG-I2** (0.015)	82
			**nG-SIPr** (0.015)	95
			**nG-SIPr-I2** (0.015)	47
5	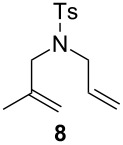	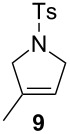	**nG** (0.05)	93
			**nG-I2** (0.05)	79
			**nG-SIPr** (0.05)	99
			**nG-SIPr-I2** (0.05)	75
6	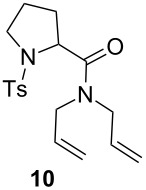	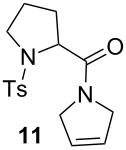	**nG** (0.04)	80
			**nG-I2** (0.04)	94
			**nG-SIPr** (0.04)	99
			**nG-SIPr-I2** (0.04)	94

^a^Toluene, 0.2 M, 70 °C, 2 h.

As outlined in Table 2, all tested catalysts were similarly effective in CM of methyl undecenoate **12** with *cis*-1,4-diacetoxy-2-butene (**13**), but parent dichloro complexes provided smaller quantities of dimerization product of **12**. In CM of **12** with electron deficient methyl acrylate **15**, diiodo derivatives were significantly less efficient and provided much more dimer of **12**. Apparently **nG-I2** and **nG-SIPr-I2** can perform noticeably better than parent dichloro complexes only in metathesis of sterically non-demanding substrates. With this knowledge, we decided to test their applicability under conditions which require high stability of the active species. Macrocyclization of dienes having low effective molarity provides access to a number of valuable musk-like compounds [27–28]. This type of transformation must be carried out at a very low concentration (usually <10 mM) in order to avoid formation of oligomeric/polymeric byproducts. Moreover, high temperature is required to complete the reaction in an acceptably short time. Therefore, a very stable and efficient catalyst is required to perform macrocyclization at reasonable loadings. The additional challenge related to high dilutions is the efficient removal of ethylene, which can be especially difficult on a large scale. Accordingly, the optimal catalyst for macrocyclization should form stable active species (usually ruthenium methylidenes), but it should also exhibit high preference of productive metathesis over unproductive metathesis.

**Table 2:** Results of CM reactions.^a^

Entry	Substrates	Product	Catalyst (mol %)	GC Yield (selectivity) [%]	*E*/*Z*

1^b^	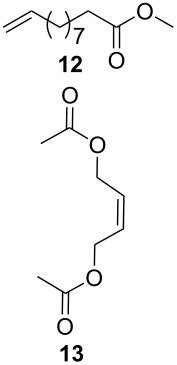	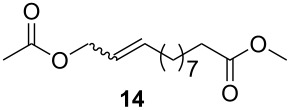	**nG** (0.4)	84 (99)	6/1
			**nG-I2** (0.4)	88 (96)	4.8/1
			**nG-SIPr** (0.4)	88 (98)	5/1
			**nG-SIPr-I2** (0.4)	84 (90)	3/1
2^c^	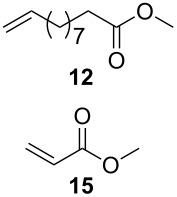	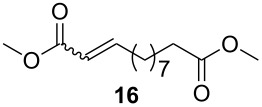	**nG** (0.5)	98 (>99)	19/1
			**nG-I2** (0.5)	74 (88)	9/1
			**nG-SIPr** (0.5)	98 (>99)	9/1
			**nG-SIPr-I2** (0.5)	30 (44)	9/1

^a^Toluene, 0.2 M, 70 °C, 2 h; ^b^3 equiv of **13**; ^c^3 equiv of **15**.

### Experiments with ethylene

**Figure 3:**
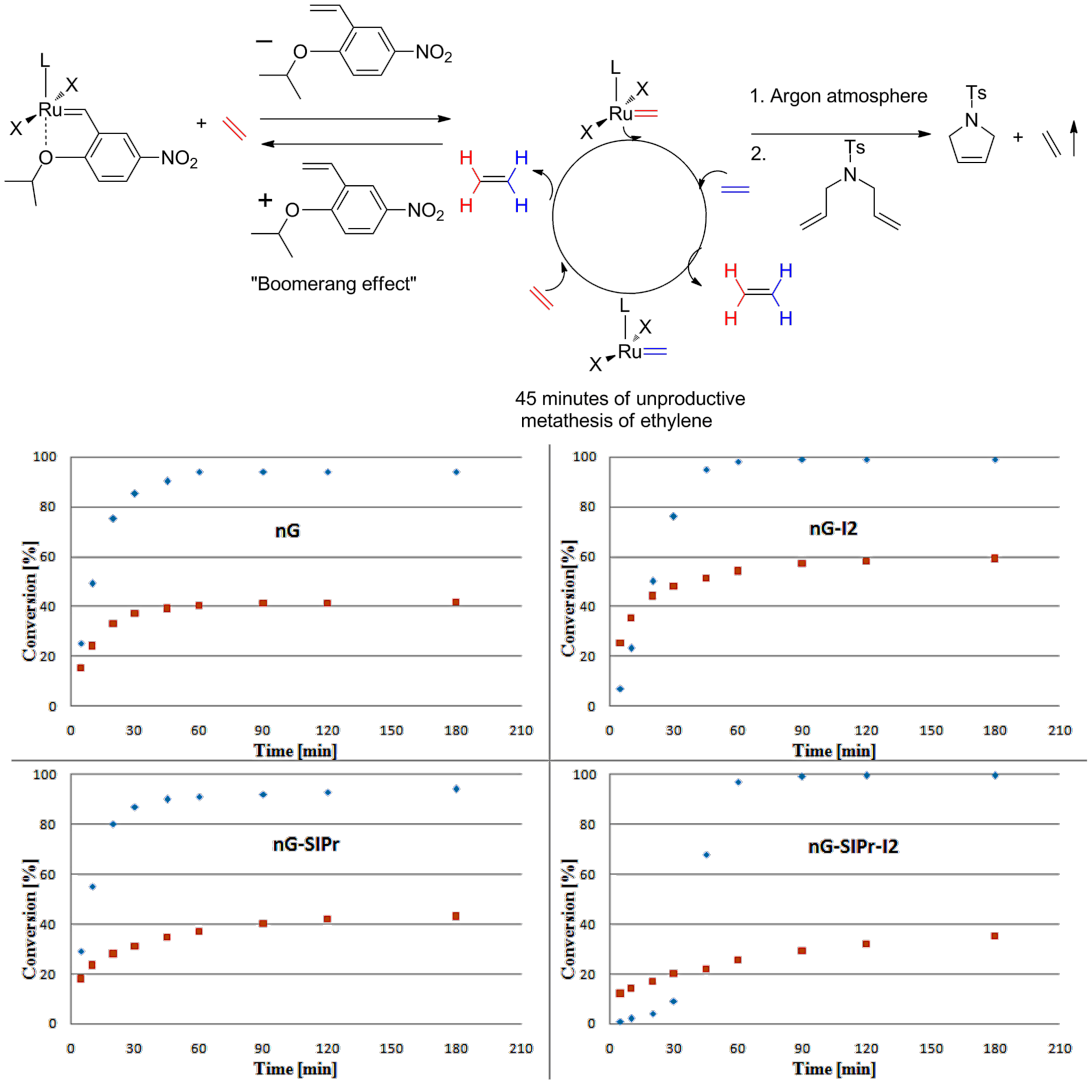
RCM of **1** (toluene, 0.05 M, 25 °C, [Ru] 0.01 mol %); blue diamonds – original (pre)catalysts; red squares – complexes pretreated with ethylene for 45 minutes.

To gain more information about the behavior of tested catalysts in the presence of ethylene, we performed two experiments. In the first test, 100 ppm of each catalyst was stirred for 45 minutes at 25 °C in an ethylene atmosphere [29–30]. During that period, ruthenium methylidenes were generated and involved in the unproductive metathesis of ethylene (Figure 3). Subsequently, the atmosphere was changed to argon and the substrate **1** (C^0^**_1_** 0.05 M) was added. To our surprise, ethylene pre-treatment had the strongest negative effect on the most sterically crowded **nG-SIPr-I2**, which in our initial tests showed the highest efficiency in RCM of **1**. In contrast, **nG-I2** turned out to be the least sensitive to ethylene. Both dichloro complexes showed similar levels of stability. These results suggest that most stable ruthenium methylidenes were generated from **nG-I2**.

**Figure 4:**
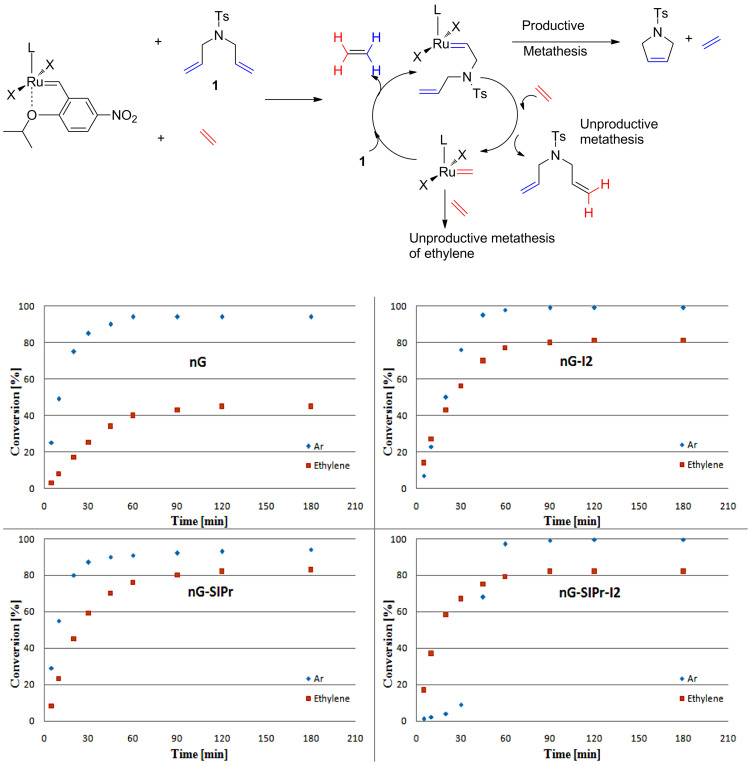
RCM of **1** (toluene, 0.05 M, 25 °C, [Ru] 0.01 mol %): top – productive RCM and possible non-productive events; bottom – reaction profiles of tested catalysts (blue diamonds – reaction under Ar, red squares – reaction under ethylene).

Next, the RCM of **1** was carried out under ethylene atmosphere which increases the probability of unproductive events (Figure 4). In this setup, the efficiency of catalysts decreased in the following order: **nG-I2** = **nG-SIPr-I2 > nG-SIPr > nG**. Good conversion obtained with **nG-SIPr-I2** indicated high preference of this catalyst toward productive RCM over non-productive metathesis. This observation partially explains the high efficiency of this catalyst obtained in RCM of **1** under conventional conditions. On the other hand, fast initiation of **nG-SIPr-I2** under ethylene suggests that in the first catalytic turn-over, the small molecule of ethylene is coordinated to the ruthenium generating highly active methylidene species.

### Macrocyclization reactions

As model substrates for macrocyclization we choose esters **17** and **18** which are metathesized to the 16- and 14- membered lactones. The RCM was run in toluene, at 70 °C and at 5 mM concentration; the catalysts were added in 10 portions with 7 minutes intervals. The 16-membered lactone **19** was synthesized with the catalyst loading of 0.3 mol % (Table 3, entries 1–4). The highest yield (91%) along with good selectivity (93%) was obtained with **nG-I2** while only a 54% yield and rather poor selectivity (70%) was observed for **nG.** Low selectivity of the reaction promoted by **nG** was the result of the formation of 13% of GC-observable byproducts (originated from double bond isomerization and ring contraction) as well as 10% of oligomeric/polymeric byproducts. The **nG-SIPr-I2** was more efficient than **nG-SIPr**, but the difference was not as striking in this pair (85% and 69% of yield, respectively). The same efficiency profile was observed in the synthesis of 14-membered **20**, which was carried out with the catalyst loading of 0.2 mol % (Table 3, entries 9–12). In this transformation each catalyst formed significant amounts of oligomeric/polymeric byproducts. Interestingly, we noticed a strong dependence of the catalyst efficiency on the argon flow over the reaction mixture which indicates the high importance of the ethylene removal in this type of RCM. The high stability of ruthenium methylidenes generated from **nG-I2** proved to be of great importance when macrocyclizations were run without active removal of ethylene (no flow of argon over the reaction mixture). In these conditions, which simulate the difficult removal of ethylene on large scale processes, **nG-I2** delivered expected products with fair yields (77% and 57% of **19** and **20**, respectively) while other catalysts demonstrated less than a 10% yield.

**Table 3:** Results of the macrocyclization reactions.

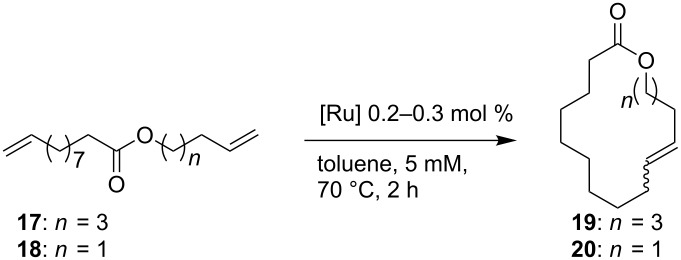					

Entry	*n*	Conditions	Catalyst (mol %)	GC Conversion (selectivity) [%]	GC Yield [%] (*E*/*Z*)

1	3	active removal of ethylene	**nG** (0.3)	77 (70)	54 (2.9/1)
2			**nG-I2** (0.3)	98 (93)	91 (3.1/1)
3			**nG-SIPr** (0.3)	77 (90)	69 (2.5/1)
4			**nG-SIPr-I2** (0.3)	90 (94)	85 (2.3/1)
5	3	no active removal of ethylene	**nG** (0.3)	5 (80)	4
6			**nG-I2** (0.3)	87 (89)	77 (2.7/1)
7			**nG-SIPr** (0.3)	8 (88)	7
8			**nG-SIPr-I2** (0.3)	7 (100)	7
9	1	active removal of ethylene	**nG** (0.2)	72 (62)	45 (8/1)
10			**nG-I2** (0.2)	99 (82)	81 (8/1)
11			**nG-SIPr** (0.2)	97 (68)	66 (9/1)
12			**nG-SIPr-I2** (0.2)	98 (73)	72 (6/1)
13	1	no active removal of ethylene	**nG** (0.2)	8 (62)	5
14			**nG-I2** (0.2)	68 (84)	57 (5/1)
15			**nG-SIPr** (0.2)	5 (60)	3
16			**nG-SIPr-I2** (0.2)	6 (50)	3

### Metathesis in ACS-grade and “green” solvents

Our continuous interest in the development of more sustainable, environmentally and user-friendly olefin metathesis has recently inspired us to test a range of commercially available, classical ruthenium initiators in ACS grade solvents under air [31]. For this study we choose substrate **1**, which is highly prone to non-metathesis reactions, namely isomerization and cycloisomerization. The result we found is that esters constitute exeptionally good solvents for RCM and CM. Conversely, application of ACS grade alcohols, ethers and toluene in many cases dramatically reduced catalyst efficiency and selectivity. It was particularly noticeable in isopropanol, in which only Hoveyda–Grubbs type complexes bearing a SIPr ligand provided expected products with 80–88% yields (0.25 mol % of catalyst, 40 or 70 °C). The catalysts containing a less sterically crowded SIMEs ligand delivered **2** with poor yield, usually accompanied by significant amounts of byproducts **21** and **22**. This demonstrates that large substituents in *N*-heterocyclic ligands (NHC) not only increased efficiency of Hoveyda-type catalysts, but also to some extent prevented formation of ruthenium species active in non-metathetical transformations.

We decided to check whether additional steric restraints around the ruthenium center caused by iodides [32] can stabilize catalysts during OM in ACS grade solvents under air. RCM of **1** carried out in toluene was accomplished by **nG** with only 54% yield and 89% selectivity (Table 4). This reduced efficiency and selectivity observed in ACS grade toluene is most probably related to the small amounts of basic amines present in this solvent [33–34]. As anticipated, **nG-SIPR** performed better, giving 92% of product and 8% of isomers. We were pleased to see that **nG-I2** and **nG-SIPr-I2** provided over 99% of the expected product. As observed previously, **nG** exhibited very low activity in 2-MeTHF while **nG-SIPR** gave 90% of **2** which was, however, accompanied by 10% of isomers. The yield (96–97%) and the selectivity (98%) for both iodide analogues were noticeably better. The advantage of sterically crowded catalysts was even more pronounced when reactions were carried out in alcohols. In iPrOH 0.075 mol % of **nG** gave only 21% of **2** with 72% selectivity; **nG-SIPr** was much more efficient (77% of yield), but the selectivity was limited (82%). In contrast **nG-I2** delivered 84% of the product with 99% selectivity, and **nG-SIPr-I2** yielded 94% of **2** with 97% selectivity. Noteworthy is that **nG-SIPr-I2** was the only catalyst able to efficiently promote RCM of **1** in methanol.

**Table 4:** RCM of **1** in ACS-grade solvents under air.^a^

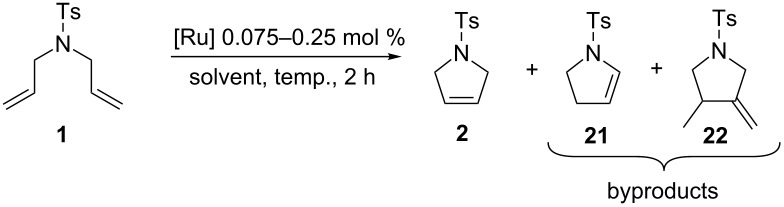				

Catalyst	GC Yield (selectivity) [%]			

	toluene^b^	2-MeTHF^c^	iPrOH^d^	MeOH^e^

**nG**	54 (89)	28 (97)	21 (72)	14 (88)
**nG-I2**	>99	97 (98)	84 (99)	47 (98)
**nG-SIPr**	92 (92)	90 (90)	77 (82)	46 (87)
**nG-SIPr-I2**	99 (99)	96 (98)	94 (97)	94 (95)

^a^Reactions carried out in non-degassed, non-distilled ACS grade solvents under air; ^b^[Ru] 0.1 mol %, 70 °C; ^c^[Ru] 0.25 mol %, 40 °C; ^d^[Ru] 0.075 mol %, 70 °C; ^e^[Ru] 0.25 mol %, 40 °C.

To further differentiate the tested catalysts, we performed RCM of DEDAM, which required an even higher stability of the active species. In this transformation, **nG** failed to give substantial amounts of the product in any solvent (Table 5). Interestingly, **nG-SIPr** exhibited very low efficiency in 2-MeTHF, but in other solvents ensured better yields than **nG-I2**. Regardless, the solvent applied, **nG-SIPr-I2**, was the most efficient catalyst.

**Table 5:** RCM of DEDAM in ACS-grade solvents under air.^a^

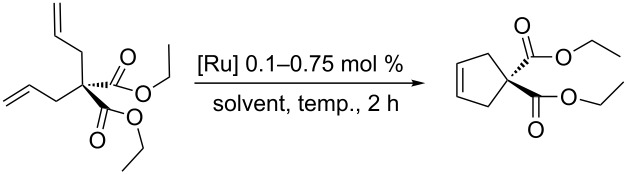				

Catalyst	GC Yield [%]			

	toluene^b^	2-MeTHF^c^	iPrOH^d^	MeOH^e^

**nG**	33	31	15	9
**nG-I2**	77	98	76	31
**nG-SIPr**	96	25	87	43
**nG-SIPr-I2**	98	100	99	64

^a^Reactions carried out in non-degassed, non-distilled ACS grade solvents under air; ^b^[Ru] 0.1 mol %, 70 °C; ^c^[Ru] 0.25 mol %, 40 °C; ^d^[Ru] 0.25 mol %, 70 °C; ^e^[Ru] 0.75 mol %, 40 °C.

In our final experiment we performed self metathesis of *tert-*butyldimethylsilyl (TBS)-protected 5-hexen-1-ol without any additives that are known to prevent double bond isomerization [35]. As expected, SM turned out to be much more challenging than RCM reactions in terms of the catalyst efficiency and selectivity (Table 6). With 1 mol % of **nG** only a minor amount of **24** was observed in toluene and no catalytic activity was noted in 2-MeTHF. **nG-SIPr** performed better in these solvents, but iodide catalysts were twice as efficient and in addition, were noticeably more selective. In alcohols 2.5 mol % of **nG** or **nG-SIPr** delivered from 9 to 19% of **24** with dramatically low selectivity in the range of 25–47%. Application of **nG-I2** or **nG-SIPr-I2** resulted in the formation of 48–72% of the expected product with fair selectivity (83–91%).

**Table 6:** CM of TBS protected 5-hexen-1-ol in ACS-grade solvents under air.^a^

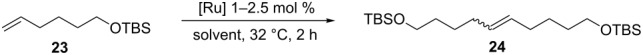				

Catalyst	GC Yield (selectivity) [%]			

	toluene^b^	2-MeTHF^b^	iPrOH^c^	MeOH^c^

**nG**	13 (87)	0	19 (27)	19 (25)
**nG-I2**	70 (99)	67 (97)	72 (91)	48 (83)
**nG-SIPr**	38 (93)	33 (87)	15 (25)	9 (47)
**nG-SIPr-I2**	67 (97)	57 (95)	65 (86)	65 (86)

^a^Reactions carried out in non-degassed, non-distilled ACS grade solvents under air; ^b^[Ru] 1 mol %; ^c^[Ru] 2.5 mol %.

## Conclusion

The iodide-containing nitro-Grela analogues exhibit improved efficiency in RCM and CM of sterically non-demanding substrates. Additional steric hindrance in the metal center proximity caused by iodides makes the 14-electron species less sensitive to small impurities, coordinative solvents (e.g., 2-MeTHF) and protic solvents. These factors lead in some cases, to dramatic improvement in the reaction(s) yield and selectivity. Increased stability of the ruthenium methylidenes generated from **nG-I2** makes this catalyst especially suitable for macrocyclization of dienes with low effective molarity.

## Supporting Information

File 1 Experimental and spectral data for **nG-I2**, **nG-SIPr-I2** and the test reactions.
