# Supplementary material for: Nitro-Grela-type complexes containing iodides – robust and selective catalysts for olefin metathesis under challenging conditions
Source: Beilstein J Org Chem. 2015 Oct 6;11:1823–32. doi: 10.3762/bjoc.11.198 (PMC4660961; doi:10.3762/bjoc.11.198)
Supplement: File 1 — Experimental and spectral data for nG-I2, nG-SIPr-I2 and the test reactions. [file Beilstein_J_Org_Chem-11-1823-s001.pdf]

Supporting Information

for

Nitro-Grela-type complexes containing iodides – robust and selective catalysts for olefin metathesis under challenging conditions.

Andrzej Tracz,[1,2] Mateusz Matczak,[1] Katarzyna Urbaniak,[1] and Krzysztof Skowerski,*[1]

Address: [1]Apeiron Synthesis SA, Duńska 9, 54-427 Wrocław, Poland and [2]Department of Bioorganic Chemistry, Faculty of Chemistry, Wroclaw University of Technology, Wybrzeże Wyspańskiego 27, 50-370 Wrocław, Poland

Email: Krzysztof Skowerski - krzysztof.skowerski@apeiron-synthesis.com

*Corresponding author

Experimental and spectral data for nG-I2, nG-SIPr-I2 and the test reactions

Contents

1. General..s2
2. Synthesis procedures and analytical data s3
3. General procedure for metathesis reactions...................................s10
4. Analytical data of metathesis products s10

1. General

Toluene was washed with citric acid (1 M in water) and deionized water, dried with sodium sulfate and by distillation over Na. Dry solvent was transferred under argon and stored over 4 Å MS. MeOH, iPrOH, and 2-MeTHF (all ACS grade) were purchased from Sigma, toluene (pure p.a.) was purchased from POCH SA (Poland); all low quality solvents were used for metathesis reactions without further purification.

Column chromatography: Merck silica gel 60 (230–400 mesh),

NMR: Spectra were recorded on Bruker Avance^{TM} 600 MHz.spectrometer and Bruker Avance III HD 500 MHz spectrometer in $CDCl_3$ or CD_2Cl_2; chemical shifts (δ) are given in parts per milion (ppm) downfield from tetramethylsilane as referenced to residual protio solvent peaks, coupling constants (*J*) in Hz.

GC: Trace GC Ultra, Thermo Electron Corporation, HP-5 column.

MS (ESI): Micromass LCT mass spectrometer and LCT PremierXE Waters mass spectrometer.

All metathesis substrates and products are known compounds. The identity of metathesis products previously reported by us and authenticated by NMR spectroscopy was confirmed by comparison of GC retention times.

Synthesis of catalysts

nG-I2

nG (30.0 g, 44.6 mmol) was suspended in MeOH (300 ml) and KI (222 g, 1.34 mol, 30 equiv) was added. The suspension was vigorously stirred for 48 h. The catalyst was filtered off, washed with MeOH and dissolved in a minimal amount of DCM. Next MeOH (300 ml) was added and DCM was evaporated. Next a portion of KI (30 equiv) was added and the exchange procedure was repeated. The crude product was purified by precipitation from a mixture of DCM/MeOH affording brown crystals, 35.5 g, 93%.

^{1}H NMR (600 MHz, $CDCl_3$) δ ppm: 15.51 (s, 1H); 8.48 (dd, 1H, J=2.4 Hz, J=9.0Hz), 7.87 (d, 1H, J=2.4 Hz); 7.14 (s, 2H); 7.01 (s, 2H); 6.94 (d, 1H, 9.0 Hz); 5.12 (hept, 1H, J=6.0 Hz); 4.18 (s, 4H); 2.67 (s, 6H); 2.52 (s, 9H); 2.34 (s, 3H); 1.47 (d, 6H, J=6.0 Hz).

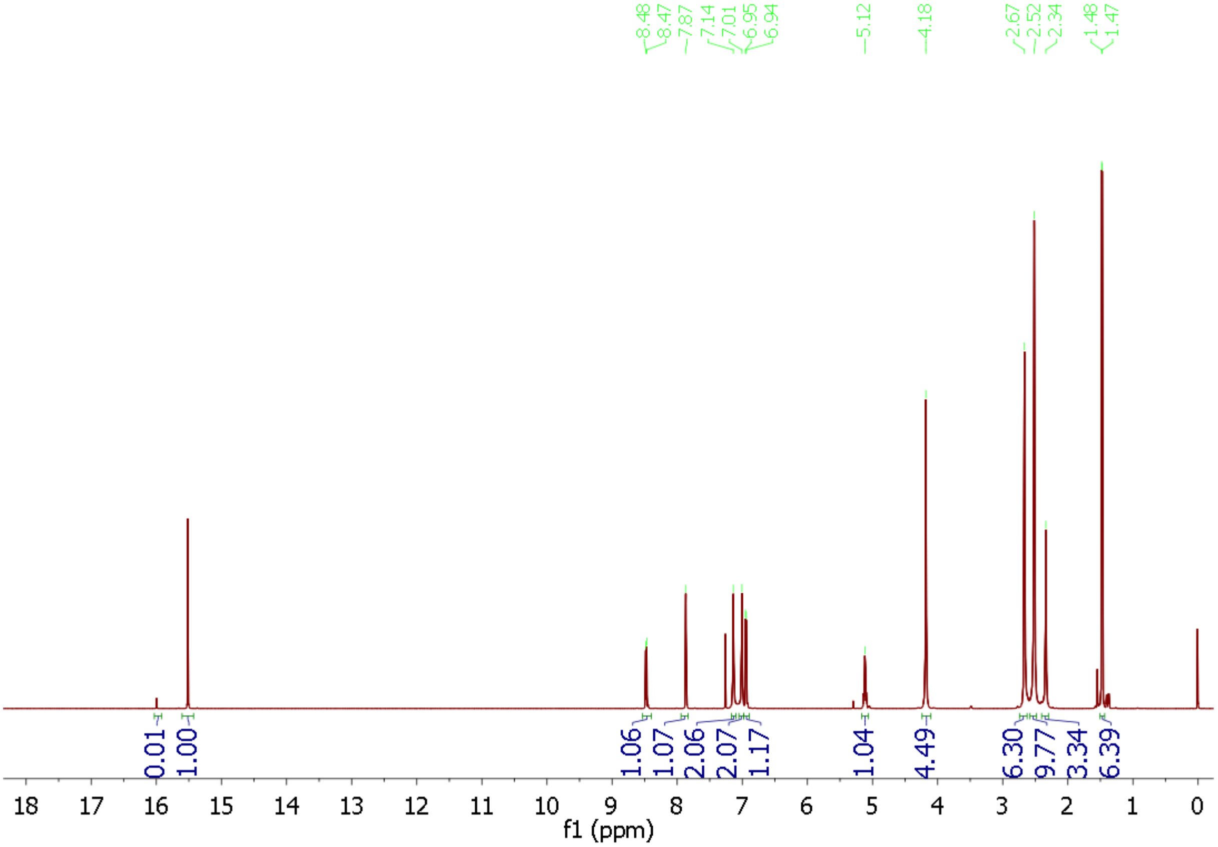

^{13}C NMR (125.4 MHz, $CDCl_3$) δ ppm: 291.7, 211.1, 156.8, 144.4, 142.7, 139.3, 138.7, 138.0, 134.2, 130.0, 129.7, 124.7, 117.9, 113.4, 78.4, 52.6, 51.2, 23.3, 21.8, 21.1, 20.0.

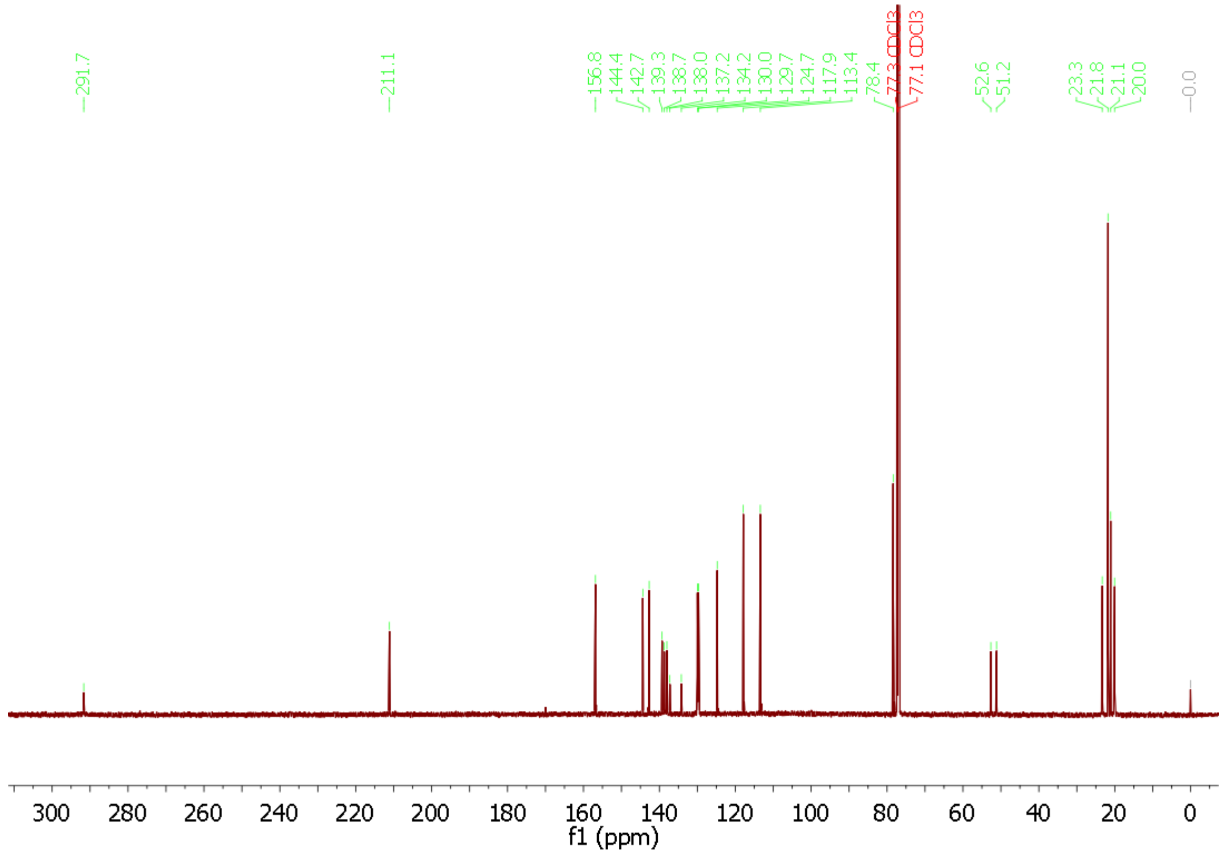

291.7
211.1
156.8
144.4
142.7
139.3
138.7
138.0
137.2
134.2
130.0
129.7
124.7
117.9
113.4
78.4
77.3 CDCl3
77.1 CDCl3
52.6
51.2
23.3
21.8
21.1
20.0
0.0
300
280
260
240
220
200
180
160
140
120
100
80
60
40
20
0
f1 (ppm)

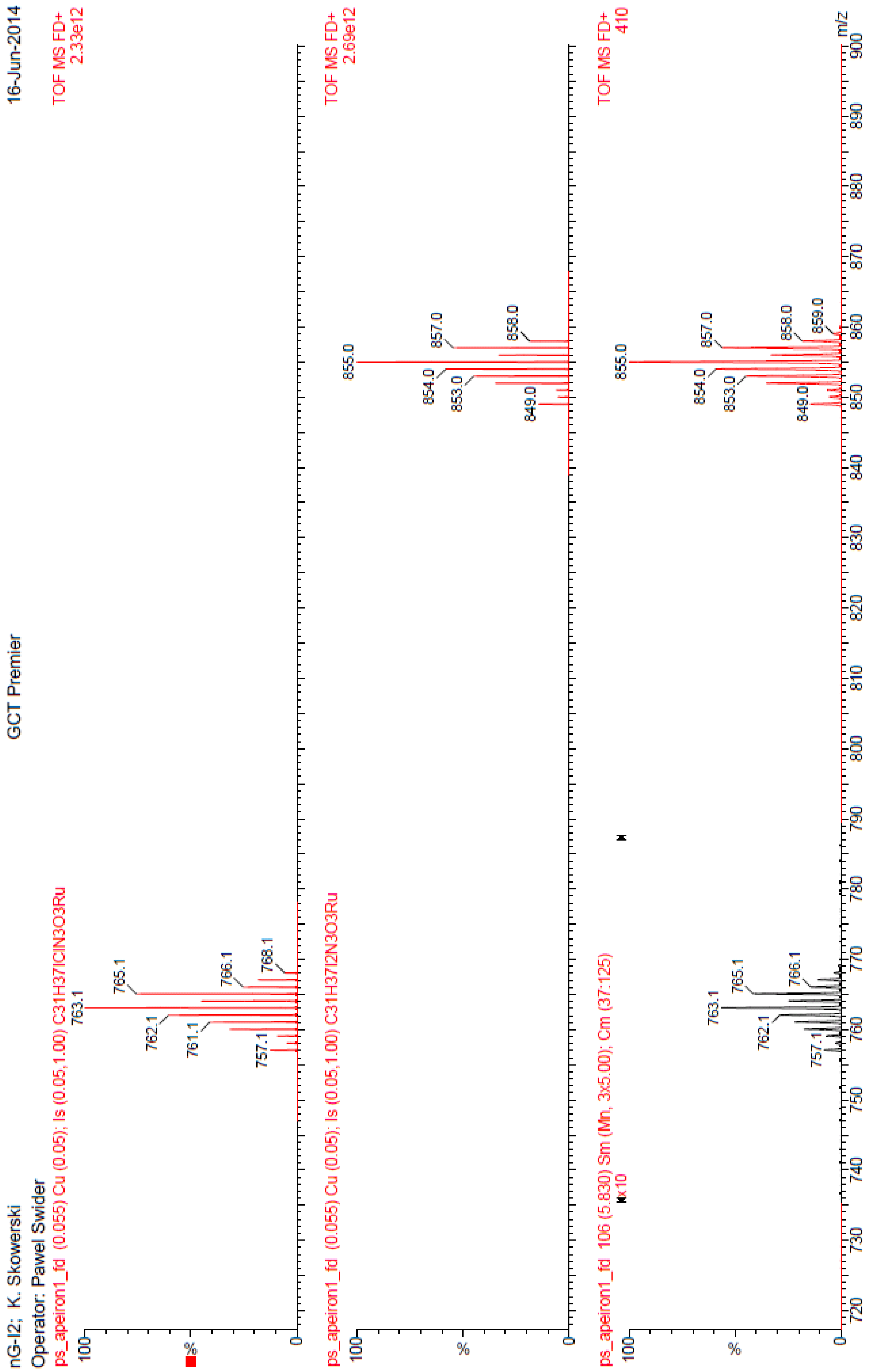

Figure S1: FD-MS spectra of **nG-I2**.

nG-SIPr-I2

nG-SIPr (45.0 g, 59.5 mmol) was suspended in MeOH (450 ml) and KI (296 g, 1.78 mol, 30 equiv) was added. The suspension was vigorously stirred for 48 h. The catalyst was filtered off, washed with MeOH and dissolved in a minimal amount of DCM. Next MeOH (450 ml) was added and DCM was evaporated. A next portion of KI (30 equiv) was added and the exchange procedure was repeated. The crude product was purified by precipitation from DCM/MeOH mixture affording dark green crystals, 52 g, 93%.

^{1}H NMR (600 MHz, CD_2Cl_2) δ ppm: 15.40 (s, 1H); 8.52 (dd, 1H, J=3.0 Hz, J=9.6Hz), 7.78 (d, 1H, J=3.0 Hz); 7.44 (bs, 6H); 7.05 (d, 1H, J=9.6 Hz); 5.23 (hept, 1H, J=6.0 Hz); 4.25 (s, 4H); 3.81 (bs, 4H); 1.66 (d, 6H, J=6.0 Hz); 1.58-1.02 (m, 24H).

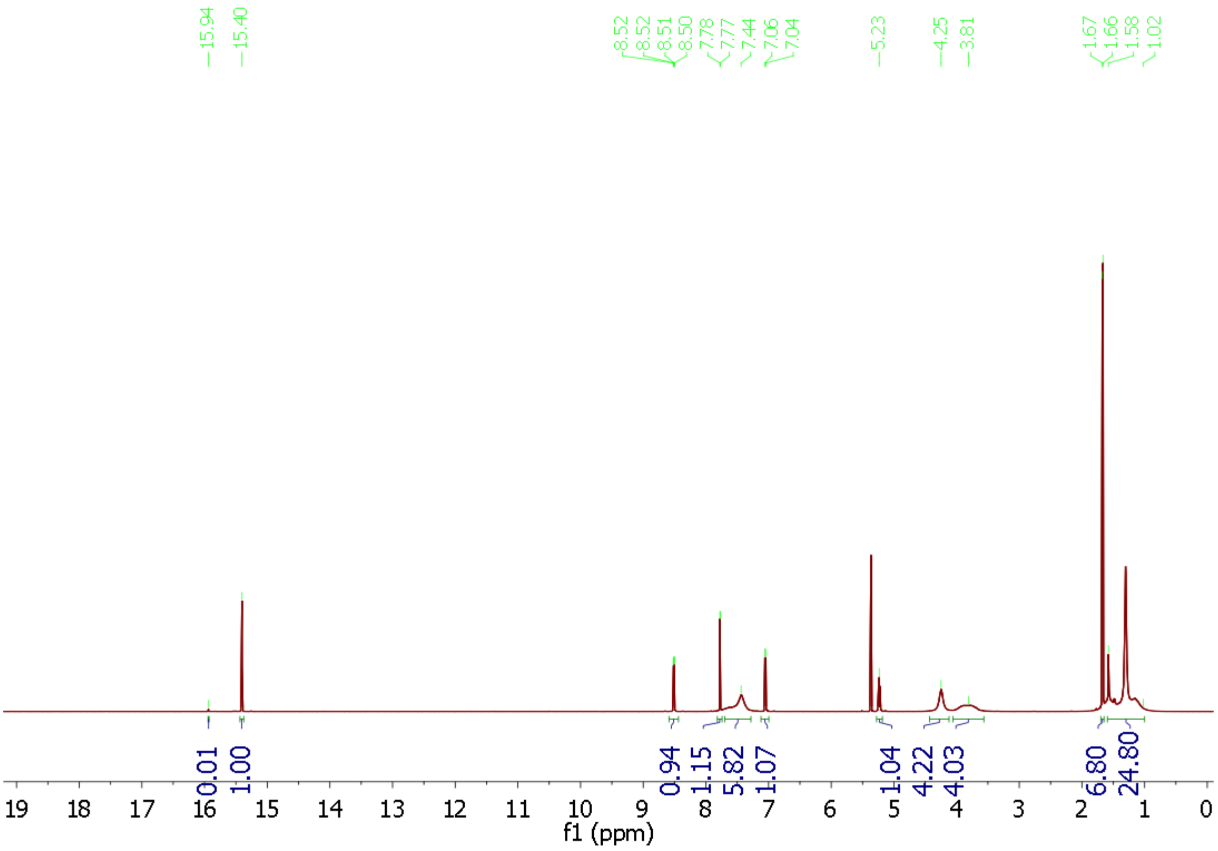

^{13}C NMR (125.4 MHz, CD_2Cl_2) δ ppm: 280.1, 214.3, 156.7, 148.8, 143.5, 142.7, 129.9, 124.5, 116.9, 113.8, 78.6, 54.9, 29.2, 28.4, 27.0, 26.2, 24.7, 23.4, 22.4.

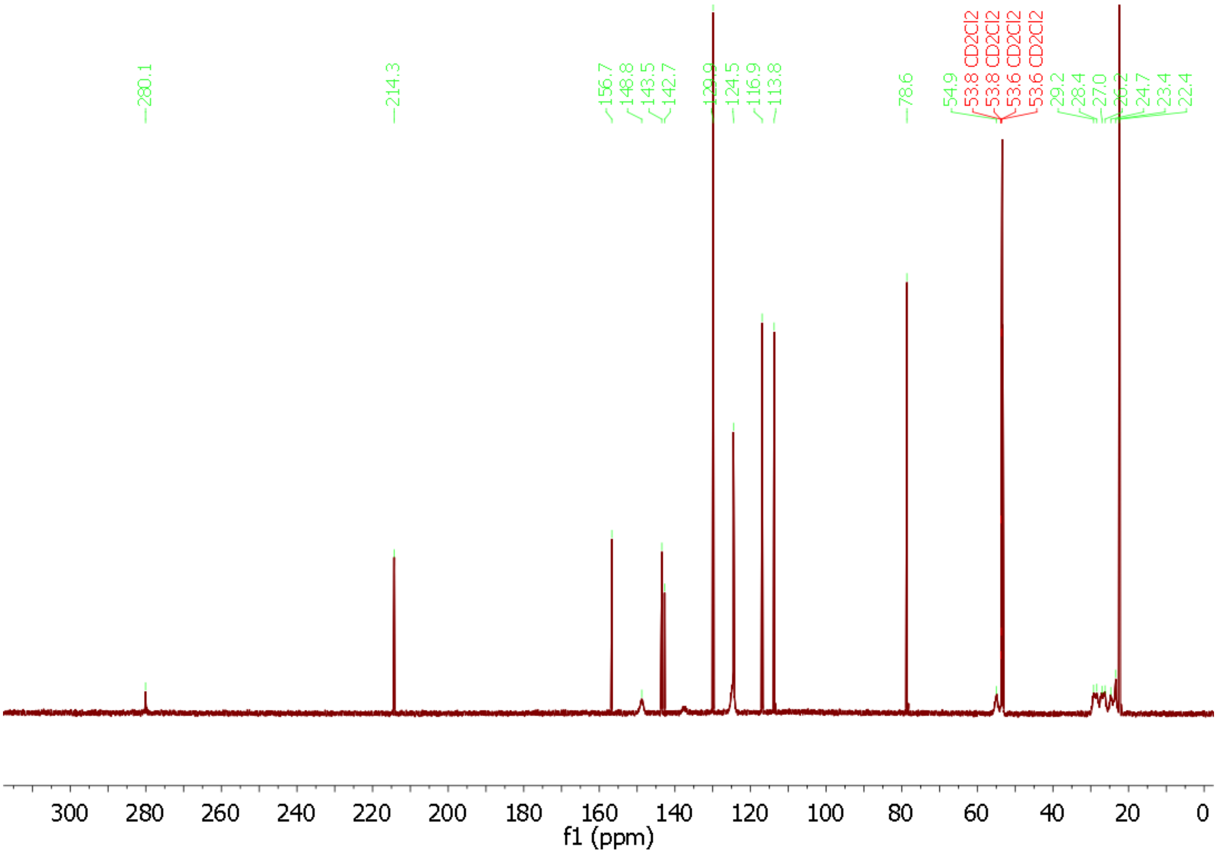
280.1
214.3
156.7
148.8
143.5
142.7
129.9
124.5
116.9
113.8
78.6
54.9
53.8 CD2Cl2
53.8 CD2Cl2
53.6 CD2Cl2
53.6 CD2Cl2
29.2
28.4
27.0
26.2
24.7
23.4
22.4
300 280 260 240 220 200 180 160 140 120 100 80 60 40 20 0
f1 (ppm)

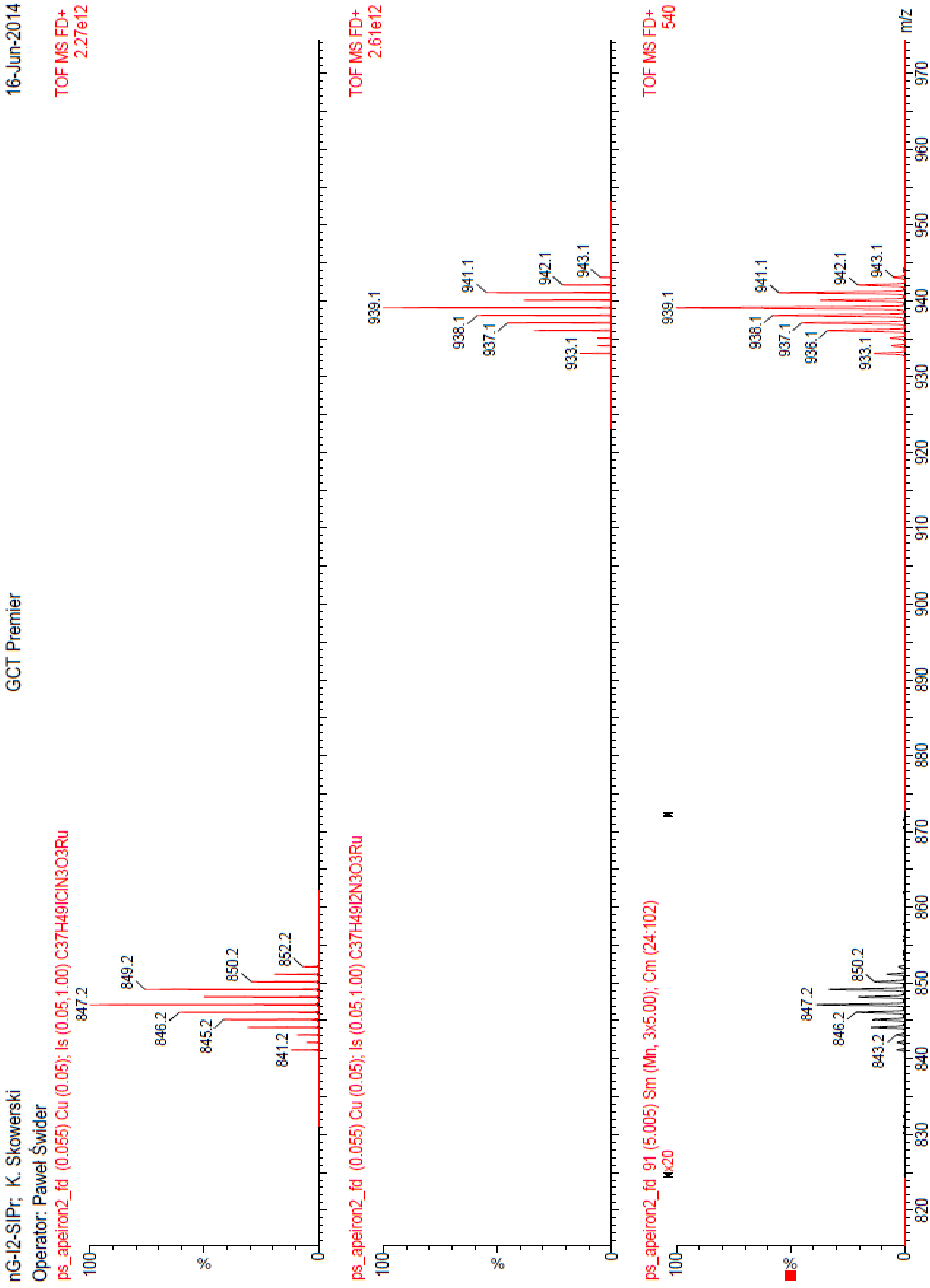

Figure S2: FD-MS spectra of **nG-SIPr-I2**.

Preparation of substrates for metathesis

N-allyl-*N*-cinnamyl-4-methylbenzenesulfonamide (**7**)

Solution of allylamine (2.99 g, 52.5 mmol) and triethylamine (8.79 ml, 62.9 mmol) in DCM (20 ml) was added to cooled (0 °C) solution of *p*-toluenesulfonyl chloride (10.0 g, 52.5 mmol) in DCM (100 ml). The reaction mixture was warmed up to room temperature and stirred for 1 h. After that reaction mixture was washed with water (3 x 25 ml) and organic fraction was dried with sodium sulfate. Evaporation of DCM provided *N*-allyl-4-methylbenzenesulfonamide (9.97 g, 47.2 mmol, 90%) as a light-yellow oil which was dissolved in DMF(80 ml). To this solution potassium carbonate (9.78 g, 70. 8 mmol) and 3-bromo-1-phenyl-1-propene (11.16 g, 56.6 mmol) were added. The Reaction mixture was stirred at 50 °C for 24 h. DMF was removed and water was added to the residue. The product was extracted with ethyl acetate (3 x 30 ml), the organic fraction was washed with water (2 x 20 ml) and dried with sodium sulphate. After removal of the solvent the crude product was purified using column chromatography (c-hexane/ethyl acetate 7/3). The product was obtained as a light yellow oil (12.36 g, 80 %) which was treated with activated alumina prior to metathesis reactions.

^{1}H NMR (500 MHz, CD_2Cl_2) δ ppm: 7.73-7.71 (m, 2H); 7.29-7.20 (m, 7H); 6.39 (d, 1H, J=16.5 Hz); 5.92 (dt, 1H, *J*=6.5 Hz, *J*=16.5 Hz); 5.68-5.60 (m, 1H); 5.17-5.13 (m, 2H); 3.95 (d, 2H, *J*=6.5 Hz); 3.84 (d, 2H, *J*=6.5 Hz); 2.41 (s, 3H).

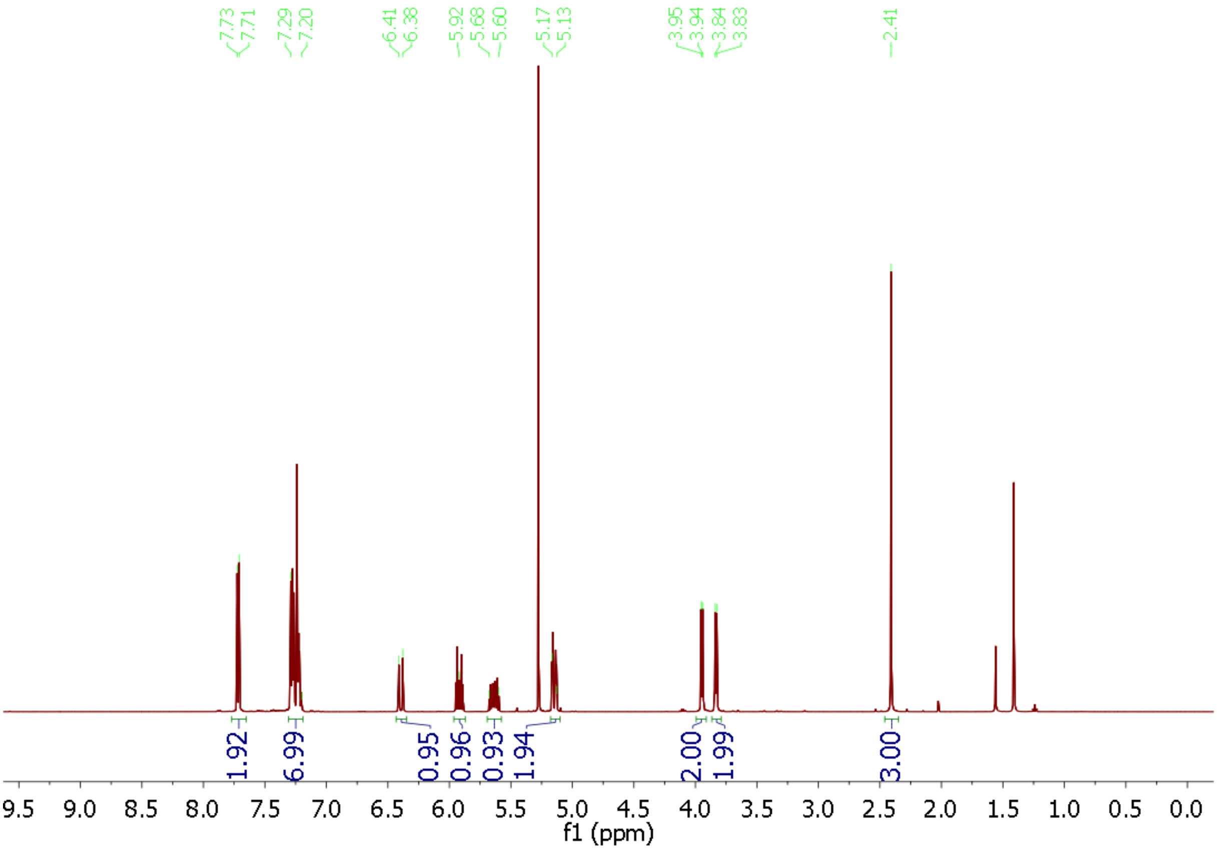

General procedure for metathesis in dry, degassed Toluene.

Substrate (1 mmol) was placed in a flame-dried Schlenk tube and dry, degassed toluene (C = 0.2 M) was added. The reaction vessel was placed in an oil bath and was thermostated at 70 °C for 15 minutes prior to addition of the solution of the catalyst in dry, degassed toluene. After 2 h a sample for GC analysis was taken and it was quenched with ethyl vinyl ether.

General procedure for macrocyclizations (in dry, degassed toluene).

A stock solution of substrate **17** or **18** in dry, degassed toluene (0.01 M, 10 ml) was placed in a flame-dried Schlenk tube and dry, degassed toluene (10 ml) was added. The reaction vessel was placed in an oil bath and it was thermostated at 70 °C for 15 minutes prior to addition of the first portion of catalyst. The catalyst was added (as a stock solution in toluene) in 10 portions in 7-minute- intervals. After 2 h a sample for GC analysis was taken and it was quenched with ethyl vinyl ether. The GC yield and selectivity was calculated using an external standard.

General procedure for metathesis in ACS grade solvents.

Substrate (1 mmol) was placed in a round-bottom flask and diluted with an appropriate solvent (C = 0.2 M). The reaction vessel was placed in an oil bath and it was thermostated at requested temperature for 15 minutes prior to addition of a solution of the catalyst in DCM. After 2 h a sample for GC analysis was taken and it was quenched with ethyl vinyl ether. External standards were used to calculate the GC yield and selectivity.

Analytical data of metathesis products.

1-Tosyl-1,2,3,6-tetrahydropyridine (**4**)

^{1}H NMR (500 MHz, $CDCl_3$) δ ppm: 7.67-7.64 (m, 2H); 7.32-7.29 (m, 2H); 5.75-5.71 (m, 1H); 5.60-5.57 (m, 1H); 3.57-3.54 (m, 2H); 3.15 (t, 2H, J=6.0 Hz); 2.41 (s, 3H); 2.21-2.17 (m, 2H).

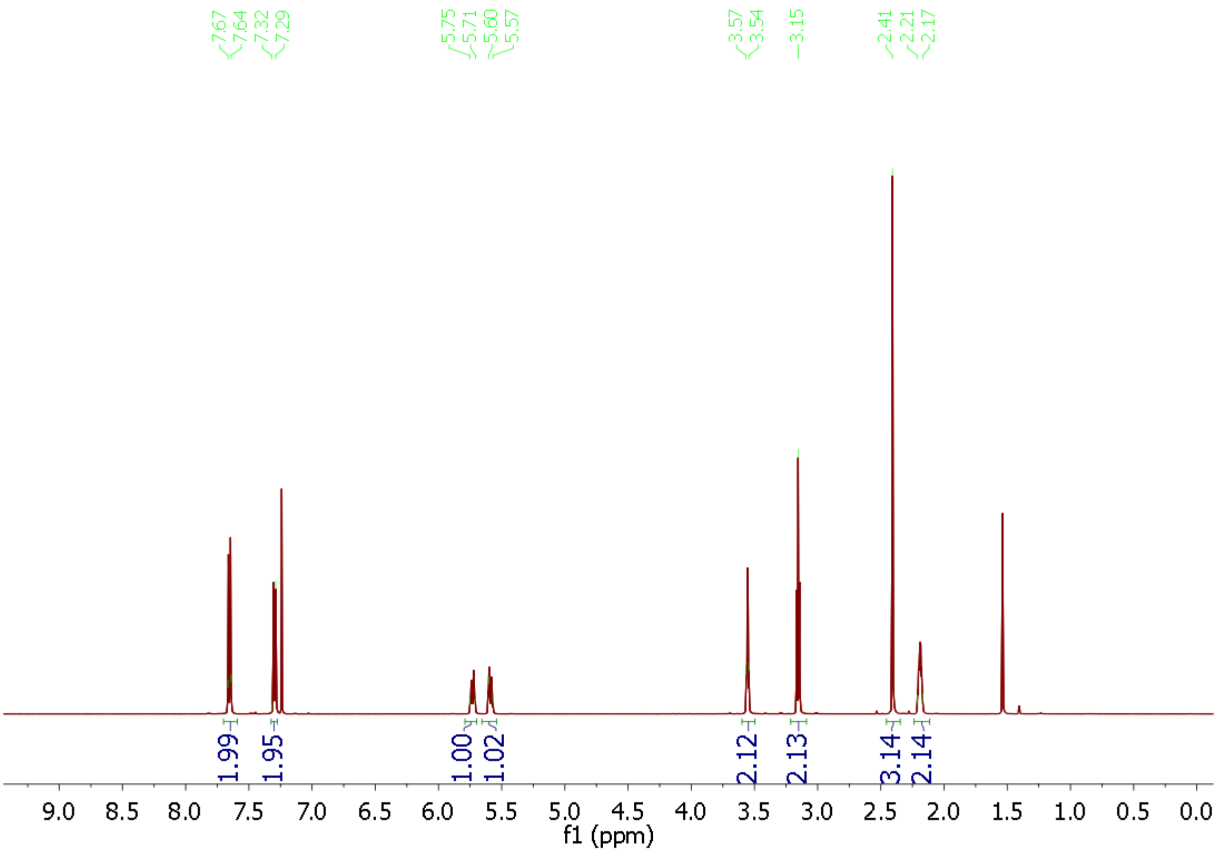

1-Tosyl-2,3,4,7-tetrahydro-1H-azepine (**6**)

^{1}H NMR (500 MHz, $CDCl_3$) δ ppm: 7.66-7.65 (m, 2H); 7.27-7.25 (m, 2H); 5.77-5.72 (m, 1H); 5.65-5.61 (m, 1H); 3.82-3.80 (m, 2H); 3.37 (*pseudo*t, 2H, J=6.0 Hz); 2.40 (s, 3H); 2.18-2.14 (m, 2H); 1.80-1.75 (m, 2H).

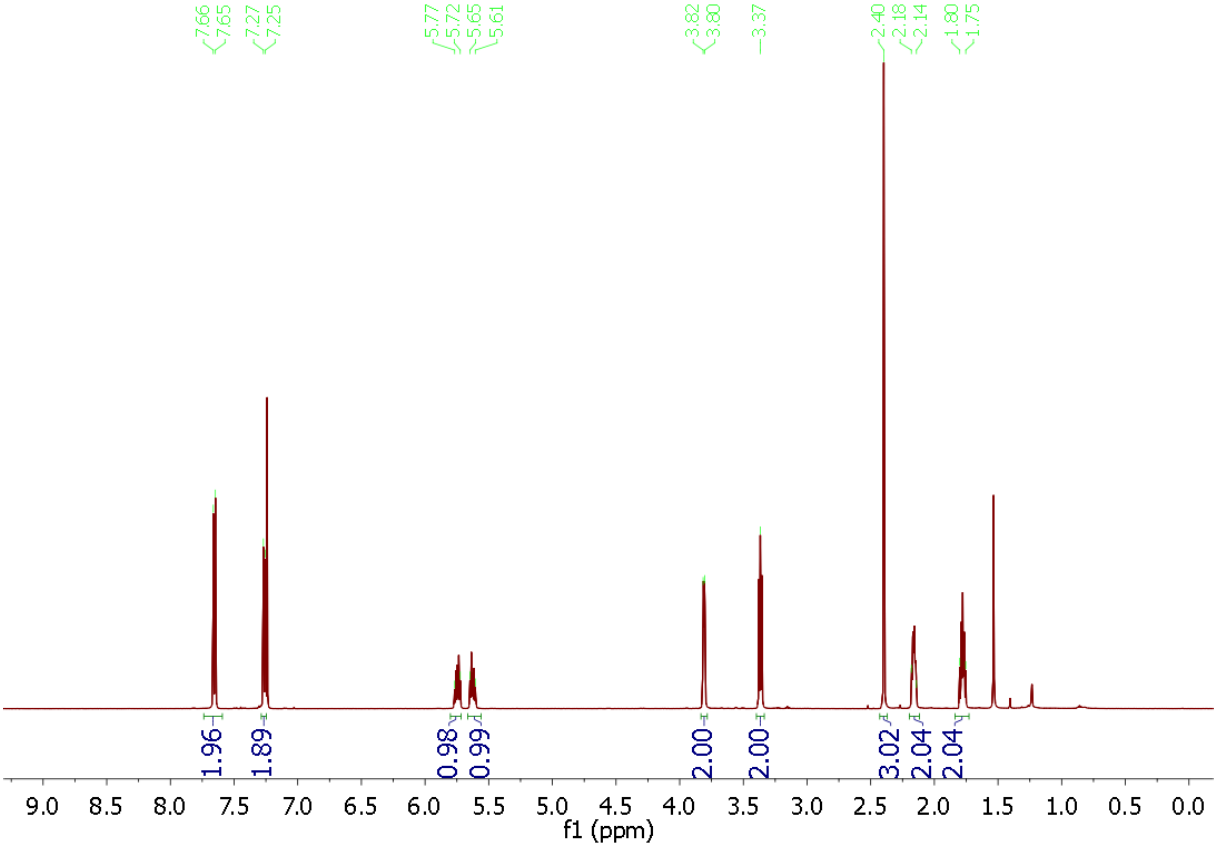

1,10-Bis-(tert-butyl-dimethyl-silanyloxy)-dec-5-ene (**24**)

E/Z 8/2; *E* isomer ^{1}H NMR (500 MHz, $CDCl_3$) δ ppm: 5.39-5.33 (m, 2H); 3.60-3.57 (m, 4H); 2.06-1.95 (m, 4H); 1.53-1.46 (m, 4H); 1.41-1.35 (m, 4H); 0.87 (s, 18H); 0.30 (s, 12H).

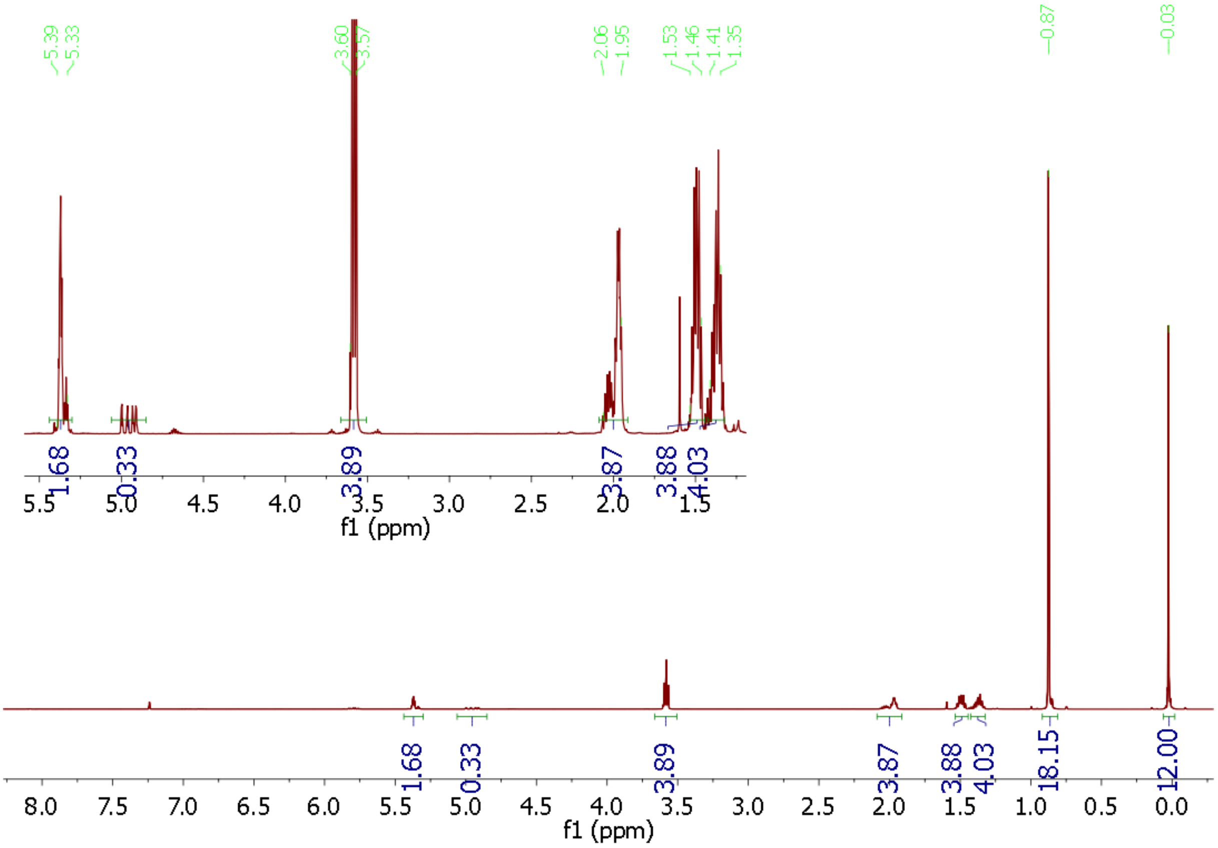
5.39
5.33
3.60
3.57
2.06
1.95
1.53
1.46
1.41
1.35
0.87
0.03
1.68
0.33
3.89
3.87
3.88
4.03
18.15
12.00
f1 (ppm)
